# The Netherlands Heart Tissue Bank: Strengthening the cardiovascular research infrastructure with an open access Cardiac Tissue Repository

**DOI:** 10.1007/s12471-022-01713-8

**Published:** 2022-07-27

**Authors:** M. T. H. M. Henkens, J. F. van Ast, A. S. J. M. te Riele, A. C. Houweling, A. S. Amin, R. Nijveldt, M. L. Antoni, X. Li, S. M. T. Wehrens, J. H. von der Thüsen, K. Damman, E. N. ter Horst, O. C. Manintveld, R. Y. Abma-Schouten, H. W. M. Niessen, H. H. W. Silljé, J. W. Jukema, P. A. Doevendans

**Affiliations:** 1 Netherlands Heart Institute (NLHI), Utrecht, The Netherlands; 2 Department of Pathology, CARIM, Maastricht University Medical Centre, Maastricht, The Netherlands; 3 Department of Cardiology, CARIM, Maastricht University Medical Centre, Maastricht, The Netherlands; 4 Department of Cardiology, Division of Heart and Lungs, University Medical Centre Utrecht, University of Utrecht, Utrecht, The Netherlands; 5 Department of Clinical Genetics, Amsterdam UMC (location VUmc), Amsterdam, The Netherlands; 6 Department of Clinical and Experimental Cardiology, Heart Failure Research Centre, Amsterdam UMC, Amsterdam, The Netherlands; 7 Department of Cardiology, Radboud University Medical Centre, Nijmegen, The Netherlands; 8 Department of Cardiology, Leiden University Medical Centre, Leiden, The Netherlands; 9 Netherlands Brain Bank, Netherlands Institute for Neuroscience, Amsterdam, The Netherlands; 10 Erasmus MC Transplant Institute, University Medical Centre Rotterdam, Rotterdam, The Netherlands; 11 Department of Cardiology, University Medical Centre Groningen, University of Groningen, Groningen, The Netherlands; 12 PLN Heart Foundation, Middenmeer, The Netherlands; 13 Department of Cardiology, Erasmus Medical Centre, Rotterdam, The Netherlands; 14 Dutch Heart Foundation, Den Haag, The Netherlands; 15 Department of Pathology and Cardiac Surgery, Amsterdam UMC, location AMC and VUmc, ACS, Amsterdam, The Netherlands; 16 Department of Pathology, Erasmus MC, University Medical Centre Rotterdam, Rotterdam, The Netherlands

**Keywords:** Heart diseases, Registries, Translational research, Tissue banks, Biological specimen banks

## Abstract

**Aim:**

Cardiac diseases remain a leading cause of cardiovascular disease (CVD) related hospitalisation and mortality. That is why research to improve our understanding of pathophysiological processes underlying cardiac diseases is of great importance. There is a strong need for healthy and diseased human cardiac tissue and related clinical data to accomplish this, since currently used animal and in vitro disease models do not fully grasp the pathophysiological processes observed in humans. This design paper describes the initiative of the Netherlands Heart Tissue Bank (NHTB) that aims to boost CVD-related research by providing an open-access biobank.

**Methods:**

The NHTB, founded in June 2020, is a non-profit biobank that collects and stores biomaterial (including but not limited to myocardial tissue and blood samples) and clinical data of individuals with and without previously known cardiac diseases. All individuals aged ≥ 18 years living in the Netherlands are eligible for inclusion as a potential future donor. The stored samples and clinical data will be available upon request for cardiovascular researchers.

**Conclusion:**

To improve the availability of cardiac tissue for cardiovascular research, the NHTB will include extensive (cardiac) biosamples, medical images, and clinical data of donors with and without a previously known cardiac disease. As such, the NHTB will function as a translational bridge to boost a wide range of cardiac disease-related fundamental and translational studies.

## Introduction

Cardiac diseases remain a leading cause of hospitalisation and mortality related to cardiovascular disease (CVD) [1, 2]. The prevalence of cardiac diseases is expected to rise even further in the coming years due to the growing occurrence of CVD-related risk factors and the ageing population [1, 3]. This makes research into improved understanding of pathophysiological processes underlying cardiac diseases of utmost importance.

To accomplish this, basic and translational research with human cardiac tissue is pivotal [4–7]. However, researchers often do not have access to these samples as there is no European centralised open-access biobank. Easy availability of human cardiac tissue and related clinical data would be an important asset for many cardiovascular researchers and would strongly improve the translation of pre-clinical findings to the clinic and vice versa.

The aim of the NHTB is to boost a wide range of cardiac disease-related fundamental and translational studies. The NHTB does this by strengthening the cardiovascular research infrastructure with an open-access non-profit biobank. The NHTB will include cardiac tissue and related clinical data from donors with and without known CVDs, which will increase our understanding of cardiac diseases during early and advanced disease development.

## Methods

The NHTB will facilitate the following: i) create a better understanding of pathological changes underlying cardiac diseases; ii) optimise (early) diagnosis of cardiac diseases by combining clinical data, imaging data, and biosamples of donors with and without known cardiac disease; iii) discover novel therapeutic targets or biomarkers to prevent, treat, or potentially cure cardiac diseases.

### Design

The NHTB (biobank-ID: bbmri-eric:ID:NL_hartenbank [8]) is a biobank including both biomaterial and clinical data which was founded in June 2020 by the Netherlands Heart Institute (NLHI: a collaboration between all the cardiology departments of the Dutch University Medical Centres). An overview of the samples collected in the biobank, the data dictionary, and the procedure for data/sample requests is available at www.hearttissuebank.nl. The study is performed in accordance with the principles of the Declaration of Helsinki [9], and the European Union General Data Protection Regulation (GDPR). An independent Medical Ethics Committee (Amsterdam, the Netherlands) has approved this biobank and related registry. All donors included in the NHTB provided written informed consent for autopsy and the use of their tissue and data pseudo-anonymised for research purposes; optional consent is asked for, among others, genetic analysis and sharing of the data outside Europe and/or with commercial companies.

### Inclusion and exclusion criteria

Individuals aged ≥ 18 years and living in the Netherlands are eligible for inclusion. Individuals will not be included in the biobank if they are unable or unwilling to provide written informed consent.

As a result, not only individuals with a previously known cardiac disease (such as ischaemic cardiomyopathy, genetic cardiomyopathy, idiopathic (dilated) cardiomyopathy) based on current knowledge [10] are included as donor, but also subjects without a known medical history of any cardiomyopathy.

### From donor registration towards scientific research

The general public is informed about the existence of the NHTB and about the registration process to become a future donor by advertisements on social media, newsletters, and by treating physicians. Currently, additional communication strategies are developed in collaboration with the Dutch Heart Foundation.

**Fig. 1:**
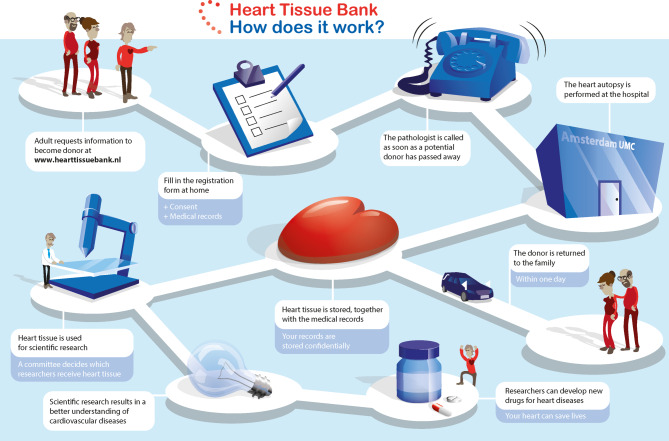
From donor registration to basic and translational research to accelerate our understanding of the development and progression of cardiac diseases and discover novel (preventive) therapeutic targets. All individuals aged ≥ 18 years living in the Netherlands are eligible for inclusion as potential future donor. Designed by Berg designs (www.bergdesigns.nl)

Every eligible subject can request the NHTB information package and registration form at www.hearttissuebank.nl. The potential future donor has the possibility to register for future donation by returning the signed registration form. Any questions related to the registration process can be answered by the NHTB team by phone or e‑mail. The future potential donor fills in a short questionnaire upon registration to give more insight into their current health status and to obtain up-to-date contact information of their treating physician(s) (Fig. 1). Cooperation of people close to the potential donor is necessary to ensure future donation; therefore, the potential future donor also appoints a confidant who signs the related registration form.

When a potential donor dies, a team, which is 24/7 available, will be called by the appointed confidant (or treating physician). The heart donation is performed, and biosamples are collected according to the standard autopsy protocol at the Amsterdam UMC, location VUmc (Amsterdam, the Netherlands), after which the body of the donor is returned to his relatives as soon as possible (Fig. 1).

Biosamples are stored within the biobank of the NHTB (Durrer Center) [11] and clinical information is stored within the electronic case report form (eCRF) after the autopsy has been performed. All stored samples and data, labelled with a unique alphanumeric code, can be used by external researchers for downstream analysis. All data and material requests will be evaluated by an independent Data Access Committee. The Data Access Committee makes recommendations for approval and rejection of access requests and ensures that the consent given by the donor aligns with the proposed research use of the material and data. The NHTB, founded in June 2020, has currently already included the first samples and expects that in 2023, 500 potential future donors will be registered and 15 NHTB autopsies will have been performed.

### Biosamples and data collection

After the heart donation, macroscopic and microscopic inspection of the heart is performed by a pathologist with cardiovascular expertise and a report of the findings will be saved in the eCRF alongside all information available related to the cause of death and timing of autopsy.

The samples will be available as frozen tissue blocks, formalin-fixed paraffin-embedded tissues, blood samples (including serum and plasma) and samples for electron microscopy (EM) analysis. Samples that currently are included in the Heart Tissue bank include blood samples from the right ventricle (stored frozen as serum and plasma), (transmural) left ventricular and right ventricular samples (as frozen tissue blocks; formalin-fixed paraffin-embedded tissues; samples for electron microscopy analysis), left/right atrial appendage and epicardial fat (as frozen tissue blocks), and transversal tissue of the pulmonary artery/veins, ascending aorta, and coronary arteries (as frozen tissue blocks). An up-to-date overview of the available samples and quality control checks are provided at www.hearttissuebank.nl. It should be stated that for certain downstream analyses, a lengthy post-mortem delay could result in significant artefacts (e.g., due to RNA degradation or proteolysis). The post-mortem delay information will be available for the researchers during sample collection requests to take this into account.

The NHTB team will request medical data from treating physicians after the autopsy is performed. These data include, but are not limited to, the medical history, adverse events and diagnostic measurements performed as part of routine clinical care (an up-to-date data dictionary, including the information requested, is available at www.hearttissuebank.nl). The diagnostic measurements include results and/or images obtained from electrocardiography, transthoracic echocardiography, computed tomography, and magnetic resonance imaging. All data collection processes are in line with the FAIR data principles [12] and Good Clinical Practice (GCP) guidelines [13]. The variables included in the database are linked to well-known ontologies (bio-ontology and SNOMED-CT).

## Collaboration with the Netherlands Brain Bank (NBB)

The Netherlands Brain Bank (NBB) and NHTB have joined forces to share facilities, expertise, expenditures and allow donors to become both brain and heart donor to further allow unravelling of the complex interplay between both organs. The NBB, founded in 1985, is a professional organisation that performed over 4500 brain autopsies and currently has over 5000 brain donors registered. The NBB has already decades of experience as a non-profit organisation in providing brain tissue (of individuals with neurological and psychiatric disorders and donors without these conditions) with extensive neuropathological and clinical data to accelerate fundamental and translational research. The NBB performs autopsies 24/7 according to standardised protocols, and together with the small population of the Netherlands, this results in a minimised post-mortem delay of, on average, 6.5 h. This results in excellent tissue quality suitable for the latest techniques. The NBB is renowned worldwide for the size and quality of its tissue and data, and the research it facilitates annually results in over hundred publications [14, 15]. Researchers can view tissue availability in an online database, and applications are evaluated by the NBB’s advisory board. All donors or their representatives provide informed consent for autopsy, storage and use of their tissue, and processing of clinical and neuropathological data for research purposes. The NBB’s procedures were approved by an independent Medical Ethics Committee (Amsterdam, the Netherlands). More information is provided at www.brainbank.nl.

## Discussion

The Netherlands Heart Tissue Bank (NHTB) is a biobank initiated to provide researchers with easy and immediate access to (cardiac) biosamples and related clinical data. The central position of the NLHI in the Netherlands, and the years of experience and joined forces with the Netherlands Brain Bank (NBB), puts the NHTB in a unique position to establish the first European open-access non-profit Heart Tissue Bank.

In the United States [16], Canada [5, 17], and Australia [6], centralised cardiac repositories are more common. However, these biobanks often do not provide clinical and imaging data during life or do not include cardiac tissue of individuals without (known) cardiac diseases. The NHTB will provide a unique source of high-quality cardiac tissues with accompanying medical data for researchers, thereby facilitating and improving the quality of cardiovascular research.

Due to its central and pioneering role in cardiovascular research in Europe, the excellent infrastructure between hospitals, the multidisciplinary collaborations between cardiologists, geneticists, pathologists and pre-clinical researchers, the years of experience of the NBB, and the close collaboration between patient organisations, health foundations and the academic community, the Netherlands is the ideal place to establish this cardiac tissue biobank. If successful, the NHTB will increase the quality and speed of cardiovascular research throughout Europe and beyond.

## Conclusion

To improve the availability of cardiac tissue for cardiovascular research, the NHTB will include extensive (cardiac) biosamples, medical images and clinical data of donors with and without previously known cardiac disease. As such, the NHTB will function as a translational bridge to boost a wide range of cardiac disease-related fundamental and translational studies.
